# Synthetic control over lattice strain in trimetallic AuCu-core Pt-shell nanoparticles†

**DOI:** 10.1039/d4nr04424j

**Published:** 2025-01-30

**Authors:** Just P. Jonasse, Marta Perxés Perich, Savannah J. Turner, Jessi E. S. van der Hoeven

**Affiliations:** a Materials Chemistry and Catalysis, Debye Institute for Nanomaterials Science, Utrecht University Universiteitsweg 99 3584 CG Utrecht The Netherlands j.e.s.vanderhoeven@uu.nl

## Abstract

Core–shell nanoparticles can exhibit strongly enhanced performances in electro-, photo- and thermal catalysis. Lattice strain plays a key role in this and is induced by the mismatch between the crystal structure of the core and the shell metal. However, investigating the impact of lattice strain has been challenging due to the lack of a material system in which lattice strain can be controlled systematically, hampering further progress in the field of core–shell catalysis. In this work, we achieve such a core–shell nanoparticle system through the colloidal synthesis of trimetallic Pt-shell Au_1−*x*_Cu_*x*_-core nanoparticles. Our seed-mediated growth methodology yields well-defined Au_1−*x*_Cu_*x*_-cores, tunable in composition from 0 at% Cu to 77 at% Cu, and monodisperse in size. Subsequent overgrowth results in uniform, epitaxially grown Pt-shells with a controlled thickness of ∼3 atomic layers. By employing a multi-technique characterization strategy combining X-ray diffraction, electron diffraction and aberration corrected electron microscopy, we unravel the atomic structure of the trimetallic system on a single nanoparticle-, ensemble- and bulk scale level, and we unambiguously demonstrate the controlled variation of strain in the Pt-shell from −3.62% compressive-, to +3.79% tensile strain, while retaining full control over all other structural characteristics of the system.

## Abstract 2

Lattice strain in trimetallic Pt-shell Au_1−*x*_Cu_*x*_-core nanoparticles was tuned from tensile strain to compressive strain by increasing the Cu fraction in the AuCu-core.

## Graphical abstract

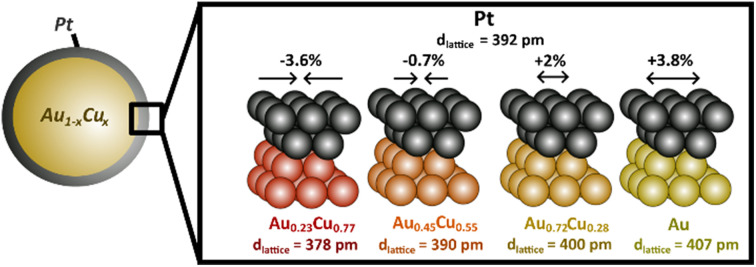

## Introduction

Core–shell nanoparticles present a versatile class of materials with highly tunable catalytic and optical properties relevant for applications in catalysis,^1–13^ plasmonics^14–18^ and sensing.^19–21^ A well-known example are core–shell nanoparticles which combine a plasmonic metal core with a catalytically active metal shell uniting the favorable optical and catalytic properties of the individual metals for plasmon enhanced catalysis and surface enhanced Raman spectroscopy.^14–17^ In the field of heterogeneous catalysis, and electrocatalysis in particular, a wide variety of core–shell materials have successfully been employed to boost catalytic reactivity and to reduce the use of precious metals, for instance, by only using the precious metal in the shell, while using a cheap, abundant metal in the core.^1^ Interestingly, core–shell nanoparticles can exhibit different and strongly enhanced catalytic performances compared to their monometallic and alloyed counterparts.^10^ As such, core–shell catalysts have rapidly been gaining ground and are being used in many different catalytic processes, ranging from electrochemical reductions^2,5–7,11,12,22^ to photocatalytic conversions^14–17^ to thermally driven oxidation^23^ and hydrogenation catalysis.^10,24,25^

To date, it is not completely understood what the origin of the altered catalytic reactivity of core–shell nanoparticles is. In literature, lattice strain and electronic effects are often invoked to explain catalytic synergy in core–shell systems.^4,13,26–33^ Lattice strain is induced in the shell material due to a lattice mismatch with the underlying core, whereas electronic effects can arise due to electronic interactions between the core with the shell material, for instance due to differences in electronegativty.^26–29,33–35^ Both effects largely depend on the choice of metals, as well as the thickness of the shell. DFT calculations suggest that electronic effects mostly impact thin shells (1–2 layers), whereas the influence of lattice strain is more dominant for thicker shells.^26,27,33,36,37^ Yet, an important bottleneck in demonstrating such effects experimentally is the lack of suitable material systems in which lattice strain can be varied controllably and independently.

Contemporary synthesis methods of core–shell nanomaterials include chemical vapor deposition, laser ablation, colloidal synthesis, or thermal annealing of a thermodynamically unfavorable mixture of metals.^38^ These methods have resulted in a wide variety of bimetallic^2,4,11,13,17,18,37,39–47^ and also trimetallic core–shell structures.^8,22,48–51^ However, intermixing between the core and the shell material, non-homogeneous shell growth and heterogeneity in particle size, shape and composition tend to complicate the resulting material systems. Furthermore, epitaxial overgrowth, where the crystal structure of the shell material matches that of the core, is an important prerequisite for achieving precise control over the shell lattice spacing, but has proven challenging to attain. Finally, simple bimetallic core–shell systems do not suffice for systematic investigations of lattice strain, since the strain is either compressive or tensile but not variable from one to the other.

In this work, we realize a trimetallic core–shell material system in which the lattice strain is varied from compressive to tensile through compositional control over the bimetallic core. Our material design relies on well-defined monodisperse trimetallic nanoparticles with an epitaxially grown Pt-shell on a Au_1−*x*_Cu_*x*_-core, where *x* indicates the fraction of Cu present in the nanoparticle core. Our seed-mediated growth strategy yields Pt/Au_1−*x*_Cu_*x*_ nanoparticles monodisperse in size and composition. Using high-resolution scanning transmission electron microscopy, electron diffraction and X-ray diffraction, we demonstrate our control over the lattice parameter in the core–shell nanoparticles at different length scales: from the individual nanoparticle scale all the way to the bulk scale.

## Results

### Seed-mediated growth of Pt/Au_1−*x*_Cu_*x*_ nanoparticles

**Fig. 1:**
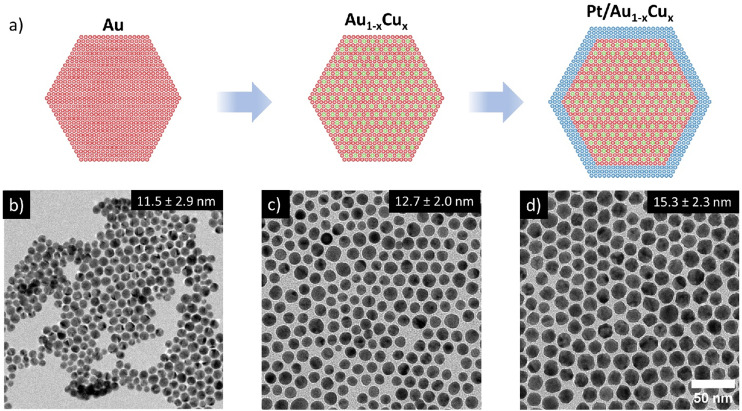
Overview of the 3-step colloid synthesis procedure used to prepare trimetallic Pt-shell Au_1−*x*_Cu_*x*_-cores. (a) Schematic overview of the applied synthesis 3-step synthesis procedure, and bright-field TEM images of (b) 11.5 ± 2.9 nm Au nuclei, (c) 12.7 ± 2.0 nm Au_0.62_Cu_0.38_ nanoparticles and (d) 15.3 ± 2.3 nm Pt/Au_0.62_Cu_0.38_ core–shell nanoparticles.

Pt/Au_1−*x*_Cu_*x*_ nanoparticles (NPs) of various compositions were successfully synthesized through a multi-step colloid synthesis process consisting of three stages, schematically depicted in Fig. 1a. First, the gold NPs were synthesized by reducing tetrachloroauric acid (HAuCl_4_) in a mixture of a high-boiling organic solvent, 1-octadecene (ODE) and two ligands, oleylamine (OAm) and oleic acid (OAc) OAm & OAc, which stabilized the metal nanoparticles in solution and helped to control the growth of the nanoparticles. Subsequently, the temperature of this mixture was raised to reduce the copper acetylacetonate (Cu(acac)_2_) precursor to form well-mixed, monodisperse Au_1−*x*_Cu_*x*_ nanoparticles. Finally, a solution of platinum acetylacetonate (Pt(acac)_2_) in OAm was added to grow an epitaxial Pt-shell, yielding well-defined Pt/Au_1−*x*_Cu_*x*_ nanoparticles. We found that adding the precursor at room temperature followed by heating to 240 °C was critical in suppressing undesired Pt NP nucleation, which does occur when introducing the Pt-precursor at elevated temperatures. Example images of the three individual growth stages of a Pt/Au_0.62_Cu_0.38_ sample are shown in Fig. 1(b–d). The bright-field transmission electron microscopy images (TEM) show that the Au nanoparticles were monodisperse and had an average diameter of 11.5 ± 2.9 nm. The NPs exhibited an intense dark red color when dispersed in toluene, which can be attributed to the localized surface plasmon resonance of the Au nanoparticles. Upon introduction of copper, the Au_0.62_Cu_0.38_ nanoparticles increased in size to an average diameter of 12.7 ± 2.0 nm, while maintaining a quasi-spherical shape (Fig. 1c) and a dark red color in dispersion. In the third and final stage of the synthesis procedure the AuCu NPs were overgrown with a Pt-shell, yielding Pt/Au_0.62_Cu_0.38_ NPs with an average size of 15.3 ± 2.3 nm (Fig. 1d) that exhibited a more facetted morphology, and had a darker brown color due to partial damping and broadening of the localized surface plasmon resonance upon Pt overgrowth.^17^ Representative UV-Vis spectra are given in the ESI (Fig. S1†). The bright field image in Fig. 1d shows contrast differences within individual nanoparticles, which are due to diffraction contrast. Diffraction contrast was observed for all compositions and indicates that the nanoparticles were polycrystalline.

### Determination of metal composition and distribution

**Fig. 2:**
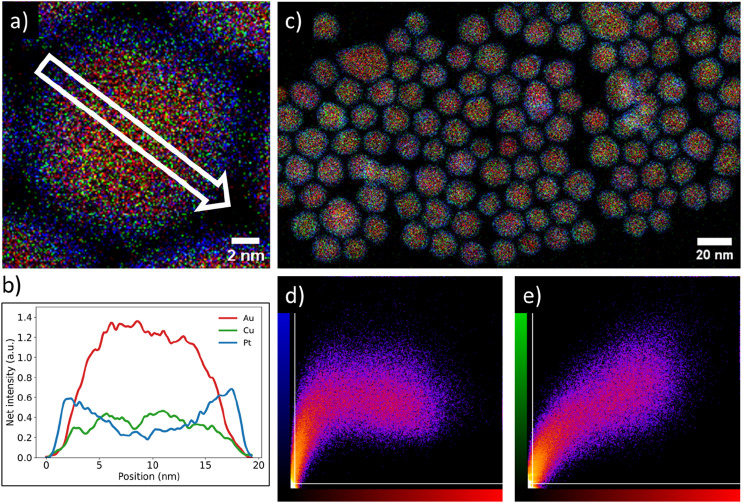
Overview of the core–shell metal distribution on both individual nanoparticle and ensemble level of a typical synthesized Pt/Au_1−*x*_Cu_*x*_ nanoparticle system. (a) Energy dispersive X-ray spectroscopy (EDX) map of a single Pt-shell AuCu-core nanoparticle indicating a core–shell metal distribution. (b) Linescan of EDX signal intensities along the white arrow drawn in (a), showing the presence of gold and copper in the core and platinum in the shell. Red, green & blue indicate gold, copper & platinum, respectively. The Pt signal was primarily located near the edge of the particle, whereas the Au and Cu signals were primarily observed in the core. (c) EDX map of Pt/Au_0.59_Cu_0.41_ nanoparticles with an average size of 15.4 ± 2.0 nm. (d) Intensity correlation plot of the Pt signal (blue) with Au signal red from (c). The brighter the observed spot, the stronger the correlation that was observed for the two signals. (e) Intensity correlation plot of the Au signal (red) with the Cu signal (green).

We verified the Pt-core AuCu-shell distribution (Fig. 2) through high resolution STEM-EDX imaging. The EDX maps in Fig. 2a and c show a single Pt/Au_0.59_Cu_0.41_ NP and several Pt/Au_0.59_Cu_0.41_ NPs, respectively. The high-resolution EDX-map in Fig. 2a clearly indicates a core–shell structure with the Au & Cu signals (red and green, respectively) located in the core and the Pt signal (blue) at the edge of the nanoparticle. A 2D-linescan (Fig. 2b) taken along the direction of the white arrow in Fig. 2a shows a 2D projection of the Au, Cu and Pt signals and confirms that the Pt signal was concentrated at the NP surface, while the Au and Cu signal were primarily located in the core and absent in the shell.

The core–shell structure as observed on a single NP was also present in a larger set of NPs. The correlation between the spatial distribution of the Au signal with the Pt and Cu signals was calculated by using a colocalization method, which provided direct insight in the average metal distribution of 147 nanoparticles. A typical EDX map used for colocalization analysis is shown in Fig. 2c along with the corresponding intensity correlation plots of the Au-to-Pt (Fig. 2d) and Au-to-Cu correlations (Fig. 2e). The intensity correlation plots depict the EDX signal intensity overlap between the Au (red) and Cu (green) or Pt (blue) signals, where brighter spots correspond to stronger overlap of signals, whereas black spots indicate no signal overlap. The white lines indicate the threshold that was applied based on the measured background in the EDX map. For a perfect correlation, a straight line from the bottom left to the top right of the graphs is expected. In the Au-to-Cu intensity correlation plot such a linear correlation is indeed observed, although higher Cu signals were also found for less intense Au signals, indicating some Cu enrichment near the edge of the core of the nanoparticle. The intensity correlation plot correlating Pt and Au signals clearly deviates from a linear trend and a similar Pt intensity was observed for varying Au intensities. Importantly, a significant number of pixels with strong Pt signal had limited Au signal. This means there were many pixels present containing Pt signal, without containing Au signal, which indicates a core–shell distribution. Combining the colocalization results with the linescan shown in Fig. 2b suggests that gold and copper were mainly located in the nanoparticle core whereas platinum was present in the shell and that this metal distribution is consistent for a large number of nanoparticles.

**Fig. 3:**
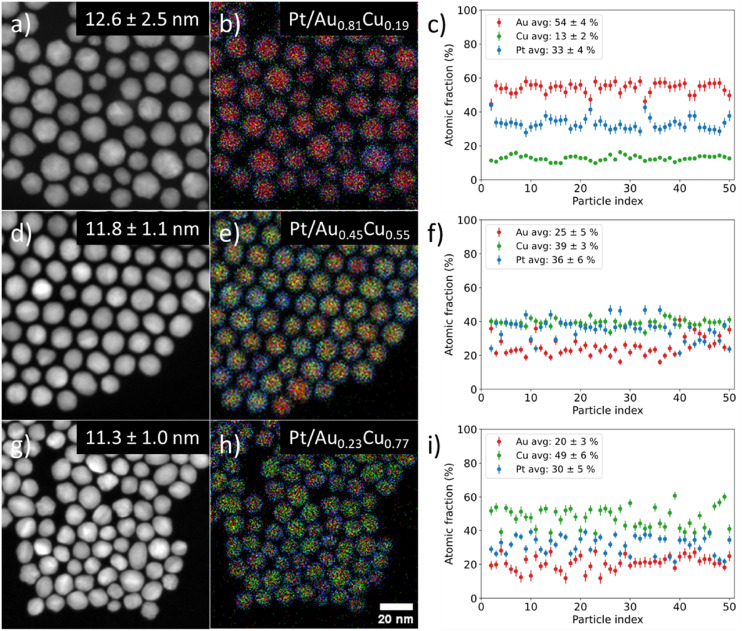
Overview of STEM-EDX investigation into the differences between individual nanoparticle compositions of three Pt/Au_1−*x*_Cu_*x*_ samples with increasing Cu core content and constant Pt composition. Here, *x* = 0.19 for (a–c), *x* = 0.55 for (d–f) and *x* = 0.77 for (g–i) show STEM images, corresponding EDX maps in (b, e and h) and individual particle compositions in (c, f and i). Red, green & blue indicate gold, copper & platinum, respectively.

We controlled the composition of the Au_1−*x*_Cu_*x*_-core over a wide range from gold- (*x* = 0.19) to copper-rich (*x* = 0.77) by varying the ratio between the metal precursors, whilst maintaining a monodisperse size and shape in all individual stages. Fig. 3 shows the STEM-EDX images for Pt/Au_1−*x*_Cu_*x*_ nanoparticles with *x* = 0.19 (a–c) 1, *x* = 0.55 (d–f) & *x* = 0.77 (g–i). The average particle size was similar; 12.6 ± 2.5 (*x* = 0.19, Fig. 3a), 11.8 ± 1.1 (*x* = 0.55, Fig. 3d) and 11.3 ± 1.0 nm (*x* = 0.77, Fig. 3h). The corresponding EDX maps in Fig. 3b, e and h show that the nanoparticles were also uniform in composition, which is further confirmed by the plots in Fig. 3c, f and i showing the atomic fraction of Au, Cu and Pt for 50 individual NPs per sample. The average atomic compositions were 54 ± 4 at% Au, 13 ± 2 at% Cu & 33 ± 4 at% Pt (Fig. 3c), 25 ± 5 at% Au, 39 ± 3 at% Cu & 36 ± 6 at% Pt (Fig. 3f) and 20 ± 3 at% Au, 49 ± 6 at% Cu & 30 ± 5 at% Pt (Fig. 3i). Only fully imaged particles were taken into account for the compositional analysis. The very low standard deviations (2–6 at%) show that in all cases the nanoparticles were highly uniform in composition. The Pt composition was used to calculate the average shell thickness (assuming a spherical shape) and was 0.76, 0.45 and 0.53 nm corresponding to 3–4, 2–3 & 2–3 Pt layers for the sample with *x* = 0.19, 0.55 and 0.77, respectively. STEM imaging used for particle size analysis, EDX analysis and size distributions of all samples presented in this work are given in the ESI (Fig. S2–S4).†

Bulk metal compositions of all colloidal suspensions were determined using ICP-OES and were in line with the STEM-EDX results. The ICP-OES results for the Pt/Au_1−*x*_Cu_*x*_ colloids with *x* = 0.19, 0.28, 0.38, 0.55 and 0.77 are shown in Table 1, along with the previously discussed EDX results and corresponding nanoparticle size distributions. Using the ICP-OES composition and assuming spherical nanoparticles with nanoparticles sizes as determined with STEM, Pt-shell thicknesses of 0.70, 0.62 & 0.56 nm corresponding to 3–4, 2–3 and 2–3 Pt layers were calculated for *x* = 0.19, *x* = 0.55 and *x* = 0.77, respectively. These values differ slightly from the values calculated based on the STEM-EDX measurements. This was probably caused by the higher Cu at% measured with ICP-OES compared to the STEM-EDX measurements. Possibly, there was still some non-reduced copper present in the suspension which was not detected by STEM-EDX, but only by ICP-OES. On the whole though, the ICP-OES and STEM-EDX results corresponded well and differed no more than a factor of 1.2 ± 0.1. The nanoparticle sizes did not directly correlate with the amount of copper added to the Au seeds. For the samples with *x* > 0.5, the ligand-to-metal ratio was larger than for the samples with *x* < 0.5, yielding smaller seeds in the latter case and thus a smaller final particle size.

**Table 1:** Overview of size distributions and elemental compositions of samples presented in this work. The numbers reported in the subscript in the sample description correspond to the composition of only the core as determined with STEM-EDX. The STEM-EDX composition was determined using at least 200 particles imaged on separate regions of the EM grids

		EDX composition (at%)			ICP-OES composition (at%)		
Sample	Size (nm)	Au	Cu	Pt	Au	Cu	Pt
Pt/Au	13.9 ± 2.4	77.0 ± 2.1	0.0 ± 0.0	23.0 ± 2.1	77.3 ± 3.9	0.0 ± 0.0	22.7 ± 2.2
Pt/Au_0.81_Cu_0.19_	12.6 ± 2.5	53.2 ± 3.3	12.5 ± 1.0	34.3 ± 3.1	54.8 ± 3.0	15.6 ± 4.0	29.6 ± 2.7
Pt/Au_0.72_Cu_0.28_	13.2 ± 2.9	47.3 ± 3.2	18.2 ± 1.3	34.5 ± 3.0	45.2 ± 1.3	21.9 ± 1.2	32.9 ± 1.1
Pt/Au_0.62_Cu_0.38_	14.4 ± 2.0	37.6 ± 2.9	22.7 ± 1.5	39.7 ± 1.1	32.9 ± 1.1	29.0 ± 2.5	38.1 ± 1.9
Pt/Au_0.45_Cu_0.55_	11.8 ± 1.1	33.9 ± 5.2	41.4 ± 5.2	24.7 ± 3.8	25.0 ± 2.4	50.0 ± 6.8	25.0 ± 3.5
Pt/Au_0.23_Cu_0.77_	11.3 ± 1.0	17.2 ± 2.5	58.6 ± 6.7	24.2 ± 3.5	15.9 ± 0.9	59.3 ± 2.5	24.8 ± 1.8

### Determination of averaged nanoparticle lattice parameter

The AuCu-core composition directly impacted the lattice parameter of the Pt/Au_1−*x*_Cu_*x*_ nanoparticles. We assessed this on different length scales through a combination of three techniques: electron diffraction, X-ray diffraction and high resolution STEM imaging. Electron diffraction was used to determine the lattice parameters for small ensembles of nanoparticles, while X-ray diffraction was used to verify these trends on a much larger, bulk scale. Local analysis of the crystal structure and interplanar spacings of the Pt-shell were assessed using HRSTEM on the single particle level.

**Fig. 4:**
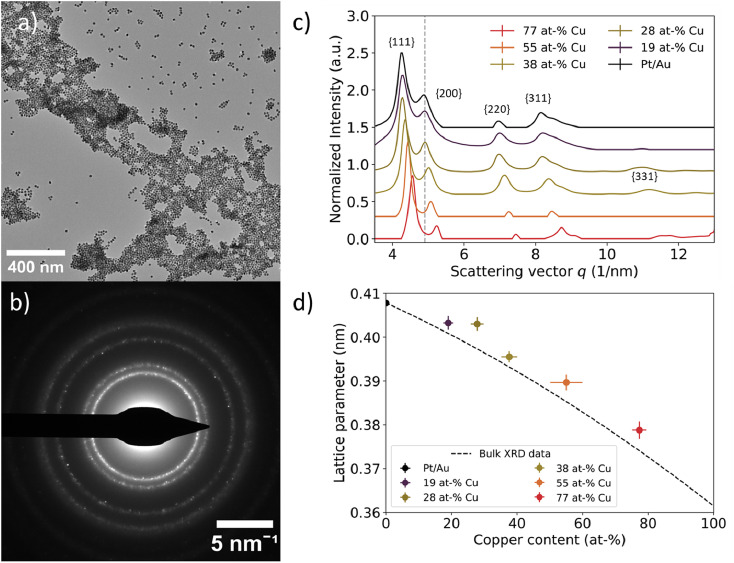
Local assessment of nanoparticle lattice parameter as a function of core Cu at%. (a) TEM image of representative area used for selected area electron diffraction. (b) Corresponding electron diffractogram at a camera length of 520 mm. The dots indicate defined crystal planes found in individual nanoparticles. (c) Azimuthally integrated intensities of 2D electron diffractograms for Pt/Au_1−*x*_Cu_*x*_ ranging from *x* = 0.19 to *x* = 0.77. The grey dotted line represents the scattering vector for the Au{200} plane family. The scattering vector increased as more copper was incorporated into the core. A vertical offset was introduced to enhance visibility, while the scattering vector remained unchanged. (d) Experimental lattice parameters plotted as function of core copper content. The lattice parameter decreased as more copper was incorporated into the core. Lattice parameters deviated approximately 1% from the corresponding literature XRD values, which are represented by the dashed black line.^52^

Selected-area electron diffraction of nanoparticle ensembles revealed a face-centered cubic (fcc) structure for all Pt/Au_1−*x*_Cu_*x*_ nanoparticles. In Fig. 4a, a typical region used for electron diffraction is shown together with the corresponding electron diffractogram in Fig. 4b. The bright spot in the middle belongs to the diffracted electron beam. From the center outwards, 4 distinct rings corresponding to the {111}, {200}, {220} and {311} plane families were observed, while the {100} and {110} plane families were absent, indicating a face-centered cubic structure. In addition, some samples also showed higher order plane reflections, including the {222} and {331} plane families (Fig. S5†), but these plane families were not abundant enough in all samples to provide resolvable diffraction spots. Overall, diffraction rings were obtained as a consequence of imaging many different colloids simultaneously with many of them lying in different crystal orientations, and the colloids being polycrystalline in nature. The rings partially consisted of individual spots corresponding to monocrystalline domains present in single colloids. An overview of all electron diffraction patterns for the samples in this work is given in the ESI (Fig. S5†).

A decreasing interplanar spacing was observed for Pt/Au_1−*x*_Cu_*x*_ with increasing Cu at% in the core. In Fig. 4c, a summary of the integrated electron diffractograms for all Pt/Au_1−*x*_Cu_*x*_ samples is shown, with *x* = 0, 0.19, 0.28, 0.38, 0.55 & *x* = 0.77. For each sample, the intensities were normalized with respect to the reflection corresponding to the {111} plane family. The peaks in Fig. 4c correspond to a diffraction ring in Fig. 4b. In all samples, reflections corresponding to the {111}, {200}, {220}, {311} plane families were observed, with decreasing intensity for higher order reflections. For each of the observed reflections, the scattering vector, defined as the distance in reciprocal nanometers (1 nm^−1^) from the center of the transmitted beam, increased with increasing copper content. This corresponds to a decreasing interplanar spacing (1/*q*) with increasing amounts of copper in the core. For Pt/Au_1−*x*_Cu_*x*_ samples with *x* = 0, 0.19, 0.28, 0.38 & *x* = 0.77, a shoulder was observed near the {311} plane family reflection, corresponding to the {222} plane family. For samples with *x* = 0.19, *x* = 0.28, *x* = 0.38 & *x* = 0.77, a reflection corresponding to the {331} plane family was also observed, while the scattering for this plane family was not observed for *x* = 0 & *x* = 0.55.

Selected-area electron diffraction of nanoparticle ensembles revealed a contracting lattice parameter for Pt/Au_1−*x*_Cu_*x*_ nanoparticles with increasing Cu content in the core. Lattice parameters for the measured samples were calculated according to eqn (2) and were plotted *versus* their core copper content in Fig. 4d. All dots with error bars represent the average and standard deviation of the lattice parameters calculated for the first 4 observed lattice plane families, {111}, {200}, {220} and {311}, for each of the measured compositions. For *x* = 0, 0.19, 0.28, 0.38, 0.55 & *x* = 0.77, the calculated lattice parameters were 0.4078 ± 0.006, 0.4042 ± 0.012, 0.4030 ± 0.0033, 0.3955 ± 0.0029, 0.3897 ± 0.002 & 0.3788 ± 0.0018 nm, respectively. The dotted black line represents the lattice parameters of compositions of disordered Au_1−*x*_Cu_*x*_ mixtures from *x* = 0 to *x* = 1, calculated from bulk XRD data available from literature.^52^ The experimentally measured lattice parameters deviated on average only 0.87% from the corresponding literature values. The lattice parameter for *x* = 0 was calculated to be slightly smaller than the literature XRD value, whereas the lattice parameters of the other 5 compositions were somewhat larger than the corresponding literature XRD values.^52^

**Fig. 5:**
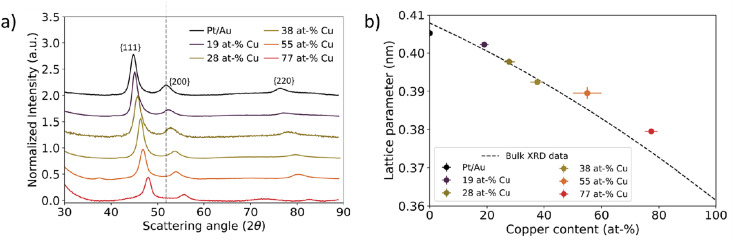
Bulk scale assessment of nanoparticle lattice parameter using X-ray diffraction as a function of Cu at%. (a) X-ray diffraction data for Pt/Au_1−*x*_Cu_*x*_ ranging from *x* = 0.19 to *x* = 0.77. The grey dotted line represents the scattering vector for the Au{200} plane family. The scattering vector increased with increasing Cu atm%. (b) Calculated lattice parameter plotted as function of core copper content. Dashed black line corresponds to XRD literature values for the Au–Cu system.^52^ In Fig. S6,† the XRD patterns for Pt/Au_0.45_Cu_0.55_ and Pt/Au_0.23_Cu_0.77_ are shown with appropriate reference patterns.

On a bulk scale, X-ray diffraction confirmed the trend of decreasing lattice parameter with increasing Cu at% in the Au_1−*x*_Cu_*x*_-core as observed with electron diffraction. Fig. 5a shows X-ray diffraction patterns of Pt/Au_1−*x*_Cu_*x*_ colloids dropcast on a Si(911) wafer, with *x* = 0, 0.19, 0.28, 0.38, 0.55 & *x* = 0.77. For all samples, a face-centered cubic crystal structure was observed following from the 3 broad reflections of the {111}, {200} & {220} plane families. The diffraction angle 2*θ* for each of these diffraction peaks shifted to higher angles with increasing copper content, indicating lattice contraction. Interestingly, the diffractogram of the *x* = 0.55 sample showed a broad and weak reflection at 37 2*θ*, which corresponds to the (110), (101) and (011) planes, which are forbidden reflections for a FCC structure. This is an indication of an ordered AuCu(i) tetragonal crystal structure, where the (110), (101) and (011) reflections are observable. The {111}, {200} & {220} plane families were used to calculate the lattice parameters shown in Fig. 5b. As expected, these lattice parameters decreased with increasing core copper content. For the samples with *x* = 0, 0.19, 0.28, 0.38, 0.55 & *x* = 0.77, the calculated lattice parameters were 0.4052 ± 0.0008, 0.4022 ± 0.0005, 0.3977 ± 0.0008, 0.3925 ± 0.0006, 0.3895 ± 0.0015 and 0.3795 ± 0.0004 nm, respectively. The lattice parameters matched the XRD literature values closely and deviated at most 0.76%.^52^

### Investigating the structure of the Pt-shell and lattice parameter

**Fig. 6:**
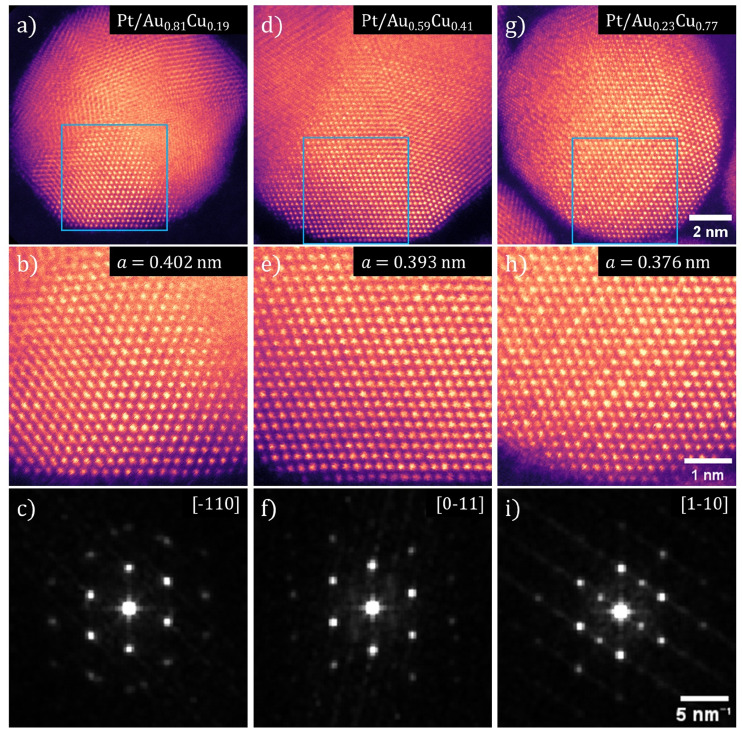
Aberration corrected high-resolution transmission electron microscopy images showing contracting lattice parameter in both Pt-shell and Au_1−*x*_Cu_*x*_-core as a function of Cu at%. HRSTEM images and corresponding FFTs for three compositions of Pt/Au_1−*x*_Cu_*x*_ nanoparticles are shown, with *x* = 0.19 for (a–c), *x* = 0.41 for (d–f) and *x* = 0.77 for (g–i). (a, d and g) Pt/Au_1−*x*_Cu_*x*_ nanoparticle with various crystals facets visible. (b, e and h) Atomically resolved region of both core and shell corresponding to the blue squares shown in (a), (d) and (g). (c, f and i) FFTs of the HRSTEM images shown in (b), (e) and (h). The zone axis indicated in the top right corner. All Pt-shells showed a deviation from the literature value for the Pt lattice parameter and demonstrate a lattice strain (when compared to literature value of 0.392 nm for Pt) of +2.42%, +0.36% and −4.03% for (d), (e) and (f), respectively.

Using HRSTEM, the lattice parameter and crystal structure of the Pt-shell within individual nanoparticles was determined. In Fig. 6, false color STEM images and corresponding FFTs of Pt/Au_1−*x*_Cu_*x*_ nanoparticles with *x* = 0.19, *x* = 0.41 and *x* = 0.77 are shown. The bright and defined spots in the STEM images show the atomic columns. The different orientation of the various crystal planes within the individual nanoparticles reveals that the NPs were polycrystalline (Fig. 6a, d and g). In the NP from the *x* = 0.19 sample (Fig. 6a) multiple crystal planes are visible that are rotated with respect to each other, which is known as crystal twinning. The twinning and polycrystallinity of the NPs made that not the entirety of the particle could be in zone axis at the same time. This out-of-zone-axis regions appear not atomically resolved. We therefore focused solely on the regions that were aligned with low order zone-axes for further analysis.

Atomically resolved regions with both shell and core in the field of view revealed that the atomic columns in the shell had the same interatomic distance as the corresponding columns in the core, which indicated that the Pt-shell had epitaxially grown on the AuCu-core and matched the lattice parameter of the Au_1−*x*_Cu_*x*_-core. The square 25 nm^2^ regions in Fig. 6b, e and h, corresponding to the blue square shown in Fig. 6a, d and g, are regions where both core and shell were visible with atomic resolution allowing an atomic scale view on the core–shell interface. The fact that the atomic columns follow the same crystal structure from core to shell, is a clear indication of epitaxial overgrowth of the Pt-shell on the AuCu-core.

The Fast-Fourier transformations (FFT) of the atomically resolved regions (Fig. 6c, f and i) confirm a contracting lattice parameter with increasing Cu at% on atomic level within a single nanoparticle, further corroborating the ensemble averaged electro- and X-ray diffraction measurements. The spots in each of the FFTs corresponds to a lattice plane of the imaged crystal with their respective indexes indicated. The spots in these FFTs corresponded well to the diffraction rings observed with electron diffraction. By resolving the FFTs using the method outlined in the Experimental section, the lattice parameter was determined for 5 images for each composition of Pt/Au_1−*x*_Cu_*x*_ nanoparticles and subsequently averaged. This resulted in a lattice parameter for Pt/Au_1−*x*_Cu_*x*_ nanoparticles with *x* = 0.19, *x* = 0.41 and *x* = 0.77 of 0.4015 ± 0.0049, 0.3934 ± 0.0066, & 0.3762 ± 0.0036 nm, respectively. Taking into account the epitaxial overgrowth of the Pt-shell as observed with HRSTEM, a lattice strain for the Pt-shell of +2.42%, +0.36% and −4.03% with respect to bulk Pt was calculated for Pt/Au_1−*x*_Cu_*x*_ with *x* = 0.19, *x* = 0.41 and *x* = 0.77, respectively.

For high Cu at% nanoparticles, atomic ordering of the Au_1−*x*_Cu_*x*_-core was observed. In Fig. 6h, spots of distinctly different contrast were observed. The slightly darker spots corresponded to atomic columns with primarily copper present. As copper is a lighter metal than either platinum or gold, its *Z*-contrast in STEM is less. Thus, atomic columns with more copper atoms appear darker than those with more platinum or gold atoms. Darker and lighter columns were regularly spaced, which is visible in Fig. 6h. This indicated that specific positions of the imaged fcc unit cells were occupied by copper and others by gold, which corresponds to an ordered AuCu_3_(i) intermetallic phase (Fig. S7†). Near the edge of the particles, this contrast difference was no longer visible. This was likely due to the fact that there was only platinum present in the shell, which indicated limited to no intermixing of copper in the shell.

Combining all lattice parameter measurements indicated that on all measured length scales, the lattice parameter contracted as a function of increasing Cu at%, showing that our Pt/Au_1−*x*_Cu_*x*_ materials are a unique nanoparticle system to controllably vary strain from tensile to compressive. A summary of all determined lattice parameters using selected area electron diffraction, X-ray diffraction and high-resolution transmission electron microscopy is given in Table 2, while corresponding shape descriptors are found in Table S1† and plotted in Fig. S8.† These shape descriptors include circularity, aspect ratio and solidity (describing surface roughness). All shape descriptors were similar and showed no clear dependence on composition. For SAED, the observed lattice parameters were on average larger than those found in literature for the corresponding composition of bulk Au_1−*x*_Cu_*x*_. The lattice parameters as determined with XRD matched literature values closely up to 38 at% Cu. Above this percentage, measured lattice parameters were again larger than those found in literature for corresponding composition of bulk disordered Au_1−*x*_Cu_*x*_. The lattice parameters determined with HRSTEM deviated the least from literature values, and these lattice parameters were on average 0.25% larger than their literature counterparts. The corresponding lattice strain was calculated according to eqn (3), using the bulk value for Pt. All in all, by increasing the Cu at% in the core from 0 at% to 77 at%, the lattice strain was tuned from +3.79% (tensile) to −3.62 (compressive).

**Table 2:** Overview of calculated lattice parameters through selected-area electron diffraction, X-ray diffraction and high-resolution scanning transmission electron microscopy. The experimentally determined lattice parameters were larger for SAED and HRSTEM measurements than for the XRD measurements. The strain (%) depicted here was calculated with eqn (3) using the average of the 3 techniques for each sample

	Lattice parameter (nm)				
Sample	Size (nm)	SAED	XRD	HRSTEM	Strain (%)
Pt/Au	13.9 ± 2.4	0.4078 ± 0.0060	0.4052 ± 0.0008	0.4089 ± 0.0038	3.79 ± 0.93
Pt/Au_0.81_Cu_0.19_	12.6 ± 2.5	0.4042 ± 0.0120	0.4022 ± 0.0005	0.4015 ± 0.0049	2.62 ± 1.40
Pt/Au_0.72_Cu_0.28_	13.2 ± 2.9	0.4030 ± 0.0033	0.3977 ± 0.0008		2.03 ± 0.69
Pt/Au_0.62_Cu_0.38_	14.4 ± 2.0	0.3955 ± 0.0029	0.3925 ± 0.0006	0.3934 ± 0.0066	0.36 ± 0.63
Pt/Au_0.45_Cu_0.55_	11.8 ± 1.1	0.3897 ± 0.0020	0.3895 ± 0.0015		−0.72 ± 0.63
Pt/Au_0.23_Cu_0.77_	11.3 ± 1.0	0.3788 ± 0.0018	0.3795 ± 0.0004	0.3762 ± 0.0036	−3.62 ± 0.51

## Discussion

This work shows for the first time the synthesis and characterization of a trimetallic Au_1−*x*_Cu_*x*_-core Pt-shell nanoparticle system of which the strain in the Pt-shell can be varied from compressive to tensile. In many cases, variation of strain in bimetallic systems is realized by changing shell thickness.^13,53,54^ As lattice strain in bimetallic core–shell structures typically decreases with increasing layer thickness,^13,54–57^ strain can be varied from the maximum strain determined by the core–shell mismatch to the minimum lattice strain determined by the thickest possible epitaxially grown shell. However, increasing shell thickness is not straightforward as island formation and dendritic growth can limit synthetic control and cause shell relaxation.^53^ In addition, using only two materials in these nanomaterials limits the nature of the lattice strain to either only compressive^3,13,31,55,58^ or tensile^3,31,32^ and not both. Probing of the entire range of strain for the same well-defined core–shell nanoparticles has only been demonstrated sparsely in literature.^4,31^ In particular, work by He *et al.* describes the controlled change of lattice strain in Pd-core Pt-shell nanocubes through a phosphorization/dephosphorization process.^4^ Like their work, we demonstrated that strain in a core–shell material can be induced in a Pt-shell by changing the underlying core structure. In contrast however, the amine and carboxylic acid ligands used in our work are relatively harmless for catalytic applications as they are easily removed through mild thermal treatments compared to the strongly binding thiols, shape-directing halides and phosphorous-based ligands typically used in the synthesis of well-defined core–shell nanoparticles.^59^ The combination of both the trimetallic nature of the presented Pt-shell Au_1−*x*_Cu_*x*_-core system and the relatively benign nature of the ligands circumvents the aforementioned issues and enables the synthesis of strained Pt-shells over a wide range of tensile and compressive strains. This makes our material system suitable for a wide range of catalytic reactions as we can target both tensile- and compressive strain-enhanced catalytic processes.^10,60–64^

The synthesized system exhibits good thermal stability and does not show signs of restructuring of the core–shell structure up to 280 °C, which is the highest temperature used during the synthesis. This is an important characteristic as core–shell structures are often prone to metal redistribution (including alloying, phase segregation and atomic ordering) at elevated temperatures, limiting their applicability to relatively mild conditions.^25,65–68^ The bulk phase diagrams of the Pt–Cu and Pt–Au system indicate the possibility of metal intermixing to some degree, with Pt and Cu forming mixed phases over the full composition range, whilst Au and Pt dissolve only slightly in each other.^52,69,70^ This makes it all the more interesting that a core–shell structure is obtained at high synthesis temperatures, also for high core Cu at%. Although STEM-EDX maps are not able to exclude core–shell intermixing on their own, colocalization analysis, line scans and atomic resolution imaging together with the STEM-EDX maps strongly suggest that Pt is located only in the shell.

Our HRSTEM results in Fig. 6 indicated that atomically ordered AuCu phases were present in some particles, which was corroborated by the XRD (Fig. 5) and ED results (Fig. 4). Atomic ordering of the crystal lattice may be expected based on previous literature on AuCu sytems.^66,71^ Atomically ordered Au_1−*x*_Cu_*x*_ phases have different lattice parameters and crystal structures compared to the disordered Au_1−*x*_Cu_*x*_ phase and can therefore induce a different strain on the Pt-shell. According to Au–Cu phase diagrams, two cubic structures, Au_3_Cu and AuCu_3_(ii), two tetragonal structures, AuCu(i) and AuCu_3_(i) and one orthorhombic structure AuCu(ii) exist.^52^ These structures have different *d* spacings. Ordered face centered cubic structures only show variations in lattice parameter below 0.5% and are thus less useful for changing lattice strain in this manner. However, a comparison between a tetragonal AuCu(i) phase and for the disordered fcc AuCu phase gives a difference of 21% for the interplanar spacing of the (111) planes (0.176 nm for the tetragonal AuCu(i) phase and a *d* spacing of 0.224 nm for the disordered fcc phase).^52^ Thus, atomic ordering of the crystal lattice opens up an additional pathway to tune Pt-shell lattice strain in our Pt/AuCu system, whilst maintaining all other structural parameters the same (core composition, particle size, Pt-shell thickness).

The Pt/AuCu material system is relevant for both fundamental and more applied studies in the fields of plasmonics and catalysis. For example, the combination of the strong plasmonic properties of the AuCu-core with the catalytic reactivity of the Pt-shell will be beneficial for plasmon enhanced catalysis^14^ and surface enhanced Raman spectroscopy (SERS) applications,^17^ in particular for *in situ* monitoring catalytic reactions.^72^*In situ* SERS has proven to be a powerful tool in gaining mechanistic insight in thermal- and electrocatalytic reactions through the spectroscopic detection of reaction intermediates.^73^ Furthermore, the tight control over Pt lattice strain in the Pt-shell Au_1−*x*_Cu_*x*_-core system could be used to demonstrate the impact of lattice strain on catalytic reactivity, and to disentangle strain effects from other effects induced by structural heterogeneities often present in core–shell systems, *e.g.* size, compositional, shell-thickness and metal distribution variations. Polydispersity in nanoparticle size can lead to variations in surface strain, in particular for small nanoparticles (<7–8 nm).^74^ Hence, the high structural control in our material system can help to exclude the influence of size effects given the relatively large nanoparticle size of our Pt-shell Au_1−*x*_Cu_*x*_-core nanoparticles (12–15 nm) and their well-defined size distributions. The synthetic control over the Pt shell thickness could be exploited to decouple strain effects from electronic core–shell effects. Such electronic effects can arise from differences in electronegativity of the shell and core metal and are typically less prominent in shells thicker than 2 atomic layers according to DFT calculations.^26,27,33,36,37^ The Pt-shell AuCu-core systems presented in this work have a shell thickness between 3 and 5 atomic Pt layers, meaning that strain effects will likely dominate over electronic core–shell effects. Theory predicts that strain can induce substantial changes in binding energy of key reactant molecules such as CO,^28,34^ oxygen^26,27^ and hydrogen.^10^ The presented Au_1−*x*_Cu_*x*_-core Pt-shell nanoparticle system enables assessing these parameters experimentally, which is directly relevant in understanding and improving the catalytic performance of core–shell catalysts for a wide range of electrocatalytic-, hydrogenation- and oxidation reactions.

## Conclusions

Altogether, this work presents a novel, trimetallic Pt-shell Au_1−*x*_Cu_*x*_-core nanoparticle system with modular lattice strain. The epitaxial overgrowth of the shell and tunable composition of core are key elements in attaining one material design in which both compressive as well as tensile strain can be induced. Our multi-scale characterization approach was critical in accurately determining the crystal structure and average lattice parameter at the bulk, ensemble and nanoparticle scale. At all length scales, the same trend prevailed: the lattice parameter decreases with increasing Cu content, and as a consequence the lattice strain in the platinum shell can systematically be varied between 3.8% and −3.6%. Our synthetic approach can be extended to other material combinations and aids the development of novel core–shell nanoparticle designs for applications in catalysis and nanomaterials science.

## Experimental methods

### Chemicals

Tetra-chloroauric acid trihydrate (HAuCl_4_·3H_2_O, ≥99.9%, Sigma Aldrich), copper(ii) acetylacetonate (Cu(acac)_2_, ≥99.9%, Sigma Aldrich), 1,2-hexadecanediol (HDD, ≥90%, Sigma Aldrich), oleylamine (OAm, ≥70%, Sigma Aldrich), oleic acid (OAc, ≥70%, Fischer Chemicals), 1-octadecene (ODE, 90%, Sigma Aldrich), toluene (Tol, ≥70%, Sigma Aldrich) were used as received. Platinum(ii) acetylacetonate (Pt(acac)_2_, ≥97%, Sigma Aldrich) was first complexed in OAm at 80 °C under N_2_ to create a 0.1 M Pt(acac)_2_in OAm solution until all Pt(acac)_2_ was dissolved and a clear yellow solution was obtained. The obtained 0.1 M Pt(acac)_2_ solution was allowed to cool down to 30 °C. All glassware was cleaned with fresh aqua regia, washed three times with Mili-Q and dried in an oven at 80 °C overnight.

### Synthesis

Pt/Au_1−*x*_Cu_*x*_-core–shell nanoparticles were synthesized through a multi-step colloid synthesis process consisting of three stages: (I) the synthesis of gold nanoparticle seeds, (II) the formation of mixed gold–copper nanoparticles and (III) the overgrowth of a platinum shell on the existing gold–copper nanoparticles. Our synthesis protocol is partially based on literature procedures by Motl *et al.*, Yang *et al.* and Khanal *et al.*^3,51,74^

In a typical experiment, 0.03 mmol of Cu(acac)_2_, 0.15 mmol of HAuCl_4_·3H_2_O and 0.42 mmol of 1,2-HDD were added to a 50 mL round-bottomed flask and dissolved in 5.00 mL ODE, 0.800 mL OAc and 0.600 mL of OAm. The specific quantities of reactants, ligands and solvent added for the different samples are listed in Table 3. (I) First the gold nanoparticle seeds were synthesized. The mixture was stirred at 400 revolutions per minute (rpm) under vacuum at room temperature (RT) for 30 min. Subsequently, it was heated to 120 °C with a heating ramp of 15 °C min^−1^ under vacuum and kept at this temperature for 30 min to remove water and oxygen and generate monodisperse Au nuclei for the subsequent synthesis steps. (II) Next, the Au_1−*x*_Cu_*x*_-cores were prepared. N_2_ was introduced and the reaction mixture was heated to 250 °C with a heating ramp of 10 °C min^−1^ whilst stirring at 400 rpm and kept at this temperature for 1 h. The copper rich samples with *x* > 0.50 were synthesized in a mixture of 15 mL OAm and 2.5 mL OAc. The mixture was first heated to 200 °C with a heating ramp of 10 °C min^−1^ whilst stirring at 400 rpm for 1 h and subsequently heated to 280 °C with a heating ramp of 10 °C min^−1^ for 1 h. In both cases, the reaction mixture was cooled down to room temperature after the final heating step whilst stirring at 400 rpm in a N_2_ atmosphere. (III) Finally, a platinum shell was grown on top of the Au_1−*x*_Cu_*x*_-cores. For the Pt overgrowth, 0.750 mL of freshly complexed 0.1 M Pt(acac)_2_ in OAm was added to the reaction mixture at 30 °C and stirred at 400 rpm under vacuum for 30 min. Next, the mixture was heated in N_2_ to 240 °C whilst stirring at 400 rpm with a heating ramp of 10 °C min^−1^ and kept at this temperature for 1 h. The mixture was then allowed to cool down to RT, after which 7.150 mL of toluene was added to the reaction mixture. Lastly, the mixture was transferred to 50 mL centrifuge vials to which 30 mL of ethanol was added before it was centrifuged at 10 000 relative centrifugal force (rcf) for 1 h. This washing procedure was repeated twice with 10 mL of toluene and 30 mL of ethanol. The mixture was redispersed in 10.00 mL of 1 vol% of OAm + OAc in toluene, sonicated for 10 min and centrifuged at 500 rcf to remove any larger aggregates. The supernatant was stored at RT in the dark.

**Table 3:** Overview of the quantities of added reactants, ligand and solvent for the samples shown in the main text

	Added reactant (mol)				Added ligand/solvent (ml)		
Sample	HAuCl_4_·3H_2_O	Cu(acac)_2_	Pt(acac)_2_	HDD	OAm	OAc	ODE
Pt/Au	0.00036	—	0.00006	—	15.00	—	
Pt/Au_0.81_Cu_0.19_	0.00015	0.00003	0.00009	0.00042	0.600	0.800	5.00
Pt/Au_0.72_Cu_0.28_	0.00012	0.00005	0.00008	0.00038	0.600	0.800	5.00
Pt/Au_0.62_Cu_0.38_	0.00007	0.00003	0.00008	0.00023	0.600	0.800	5.00
Pt/Au_0.45_Cu_0.55_	0.00012	0.00012	0.00008	0.00055	7.570	1.260	—
Pt/Au_0.23_Cu_0.77_	0.00012	0.00033	0.00014	0.00109	15.05	2.480	—

Bimetallic Au-core Pt-shell nanoparticles were synthesized according to a modified literature procedure.^3^ Here, 0.35 mmol HAuCl_4_·3H_2_O was added to a 50 mL round-bottomed flask and 15 mL OAm was added. The mixture was stirred at 400 RPM under vacuum at RT for 30 min. Next, it was heated to 120 °C with a heating ramp of 15 °C min^−1^ and kept at this temperature for 1 h under vacuum. The mixture was then brought under N_2_ and kept at 120 °C for an additional hour, after which it was cooled down to RT and 0.06 mmol of Pt(acac)_2_ was added. The mixture was then heated to 240 °C in a N_2_ atmosphere whilst stirring at 400 rpm with a heating ramp of 10 °C min^−1^ and kept at this temperature for 1 h. The mixture was allowed to cool down to RT, after which 10 mL of toluene was added to the reaction mixture. Lastly, the mixture was transferred to 50 mL centrifuge vials to which 30 mL of ethanol was added before it was centrifuged at 10 000 rcf for 1 h. This washing procedure was repeated twice with 10 mL of toluene and 30 mL of ethanol. The mixture was redispersed in 10.00 mL of 1 vol% of OAm + OAc in toluene, sonicated for 10 min and centrifuged at 500 rcf to remove any larger aggregates. The supernatant was stored at RT in the dark.

### Characterization

#### X-ray diffraction

X-ray diffraction (XRD) experiments were performed using a Bruker D2 Phaser with a CoKα-source with an X-ray wavelength of 0.17902 nm operating at 200 W with a fixed slit of 1.0 mm, an anti-scatter screen of 2.0 mm and a PSD opening of 4°. Diffractograms were acquired from 15 to 90 2*θ* with a step size of 0.1 2*θ* and a measurement time of 1 s per 2*θ* step with a rotation of 15° min^−1^. The signal-to-noise ratio was improved by measuring over the entire 2*θ* range 80 times. All measurements were performed under ambient conditions. The samples were prepared by dropcasting the concentrated colloidal suspensions on a Si(911) surface and allowing the suspension to dry at room temperature. The lattice parameters for the {111}, {200} & {220} plane families were calculated with eqn (1), the formula for the lattice parameter for FCC crystal structures:1
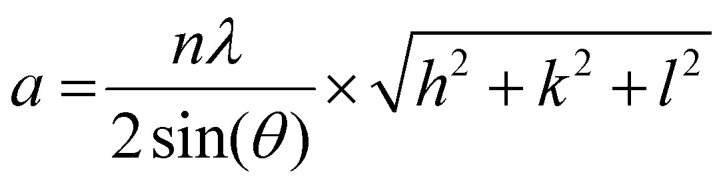


Here, *n* is the order of diffraction, *θ* is the angle of diffraction, *λ* is the X-ray source wavelength and *h*, *k* & *l* are the miller indices. The reported lattice parameters are the averaged values of the individual lattice parameters found for the {111}, {200} and {220} plane families for each sample.

#### Electron microscopy

(Scanning) Transmission Electron Microscopy ((S)TEM) images, electron diffractograms (ED) and energy dispersive X-ray spectroscopy (EDX) elemental maps were acquired with a Talos200X microscope (Thermos Fisher Scientific) operated at 200 kV. (S)TEM(-EDX) samples were measured on Carbon Type-B, 200 mesh Hex, Molybdenum grids from Ted Pella. STEM-EDX maps were acquired in a 512 × 512 px region with a pixel size of 0.512 nm and a dwell time of 5.00 μs px^−1^ with a total measurement time of 20 minutes, a collection area of 21–126 mrad, a beam convergence angle of 10.5 mrad and a typical screen current of 0.900 nA with a camera length of 260 mm. Colocalization was performed using the BIOP JACoP plugin for ImageJ. In this method, the intensity of the Au, Cu and Pt signals was compared at each pixel to determine their correlation in EDX maps. A manual background threshold was determined by evaluating the counts for each of the signals in a region of an EDX map that contained no particles. Pearson's correlation coefficients and Manders split coefficients were determined from EDX maps of 512 × 512 px regions with a pixel size of 0.512 nm.

Selected area electron diffraction (SAED) patterns were acquired with a selected area aperture size of 200 μm with a camera length of 520 mm on a CCD camera for a 4096 × 4096 pixel region with a camera exposure time of 0.1 s and a typical screen current of 0.045 nA. In all cases, a beam stopper was inserted to prevent damage to the CCD camera. 2D diffractograms were analyzed using CrysTBox software.^75^ Lattice parameters were calculated with eqn (2) for the {111}, {200}, {220} & {311} plane families, the formula for the lattice parameter for FCC crystal structures:2
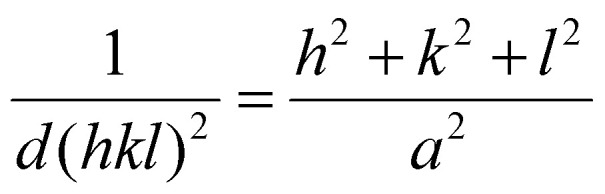


Here, *d*(*hkl*)^2^ is the interplanar spacing with indices (*hkl*), *h*, *k* and *l* are the miller indices of a specific plane and *a* is the lattice parameter. The reported lattice parameters are the averaged values of the individual lattice parameters found for the {111}, {200}, {220} & {311} plane families for each sample.

Atomic resolution STEM images were acquired with an aberration-corrected Spectra300 microscope operating at 300 kV. Images acquired had an image size of 2048 × 2048 px with a pixel size of 9.1 pm px^−1^ at a beam convergence angle of 20.6 mrad, a collection angle range of 41–200 mrad, a typical screen current of 0.150 nA and a camera length of 145 mm. The FFTs of the atomically resolved image were analyzed using the diffractGUI module from CrysTbox software.^75^ First, the software fits a 2D Gaussian to the centers of FastFourier Transform (FFT). Then, random sample consensus (RANSAC) was used to fit a regular reciprocal lattice to the set of most intense spots found in the FFT. This yields a set of basic vectors. Finally, CrysTBox maps the theoretical *d*-spacings (based on literature values for specific values of atomic fractions *x* in Au_1−*x*_Cu_*x*_) and interplanar angles to the experimental values found in the FFT of the image. In this way, the potential zone axes were determined for each image. Individual lattice parameters for each identified vector were calculated according to eqn (2). For each image, an average lattice parameter of the 4 identified vectors was calculated. The lattice strain was calculated as a percentage difference from the bulk value for platinum (*a* = 0.39239 ^76^) according to eqn (3):3
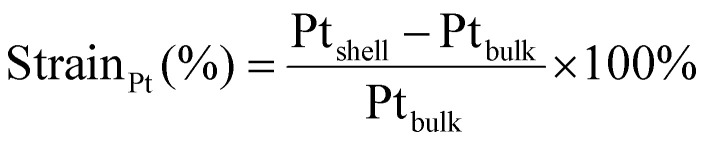


#### ICP-OES

Inductively-coupled plasma optical emission spectroscopy (ICP-OES) was performed on a Optima 8300 ICP-OES with a PerkinElmer S23 autosampler running on Syngistix software. Before and after each set of measurements, calibration curves for both Pt and Au, as well as Cu were run with concentrations of 0, 0.2, 0.4, 0.6, 0.8 and 1 mg kg^−1^. The concentrations of Pt, Au and Cu were evaluated using measured intensities at *λ* = 214.423, 299.797 & 265.945 nm, *λ* = 267.595, 242.795 & 208.209 nm and *λ* = 327.393, 222.778, 324.752 nm, respectively.

## Author contributions

J.P.J. performed the experiments supervised by J.v.d.H. M.P.P. contributed through valuable discussions and S.J.T. through her assistance in the HR-STEM measurements. J.P.J. and J.v.d.H. wrote the paper with contributions of all authors.

## Data availability

The data supporting this article have been included as part of the ESI.† Our raw data can be obtained from a dataverse repository at https://doi.org/10.34894/7M9Q6K.

## Conflicts of interest

The authors declare no competing financial interest.

## Supplementary Material

NR-017-D4NR04424J-s001
