# Supplementary material for: Synthetic control over lattice strain in trimetallic AuCu-core Pt-shell nanoparticles
Source: Nanoscale. 2025 Jan 30;17(12):7100–13. doi: 10.1039/d4nr04424j (PMC11793081; doi:10.1039/d4nr04424j)
Supplement: NR-017-D4NR04424J-s001 [file NR-017-D4NR04424J-s001.pdf]

Supplementary Information (SI) for Nanoscale.
This journal is © The Royal Society of Chemistry 2025

Synthetic control over lattice strain in trimetallic AuCu-core Pt-shell nanoparticles

Just P. Jonasse, Marta Perxés Perich, Savannah J. Turner, Jessi E. S. van der Hoeven*

Materials Chemistry and Catalysis, Debye Institute for Nanomaterials Science, Utrecht University, Universiteitsweg 99, 3584 CG Utrecht, The Netherlands

Keywords: core-shell nanoparticles, trimetallic, platinum, gold, copper, strain, electron microscopy

Optical properties of colloidal dispersions. In Figure S1, pictures and UV-Vis spectra of representative colloidal AuCu-core and Pt-shell AuCu-core samples are shown. These images show clearly that the deep red color of the AuCu-core colloidal suspension is replaced by a brown/blackish color upon Pt-overgrowth. This was caused by the dampening and broadening of the localized surface plasmon resonance (LSPR) of the AuCu-core[1].

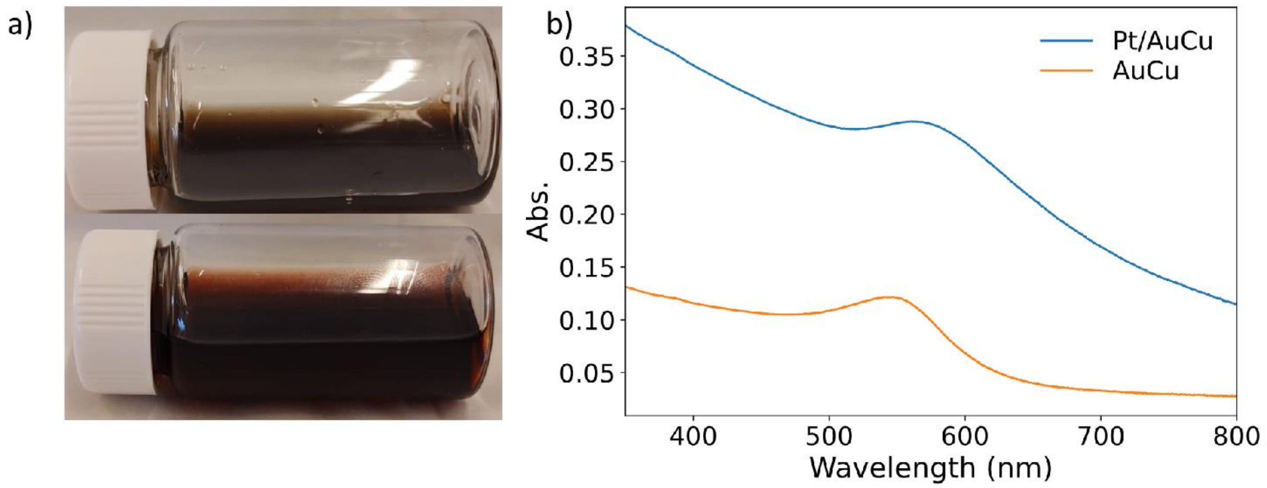

Figure S1: Optical investigation of localized surface plasmon resonance upon Pt-overgrowth. a) Picture of representative samples after Pt-overgrowth (top) and before Pt-overgrowth (bottom). b) Representative UV-Vis spectra of AuCu-core and Pt-shell (blue) and corresponding AuCu-core (orange) nanoparticle suspension in toluene.

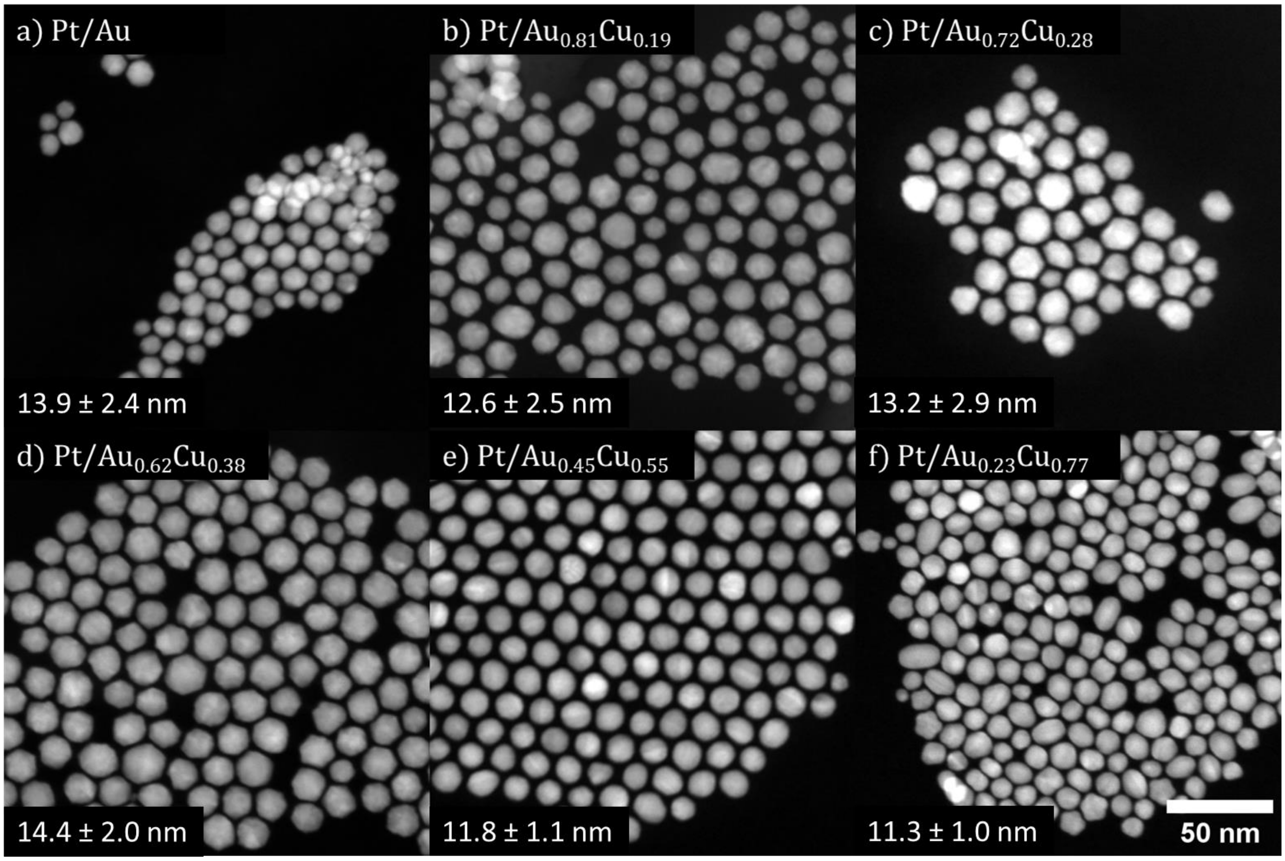

Figure S2: Scanning transmission electron microscopy (STEM) images of Pt-shell $Au_{1-x}Cu_x$-core samples reported on in this work for x = 0 (a), x = 0.19 (b), x = 0.28 (c), x = 0.38 (d), x = 0.55 (e) and x = 0. 77 (f). Scale bar corresponds to all images. Nanoparticle sizes are based on at least 200 counted particles.

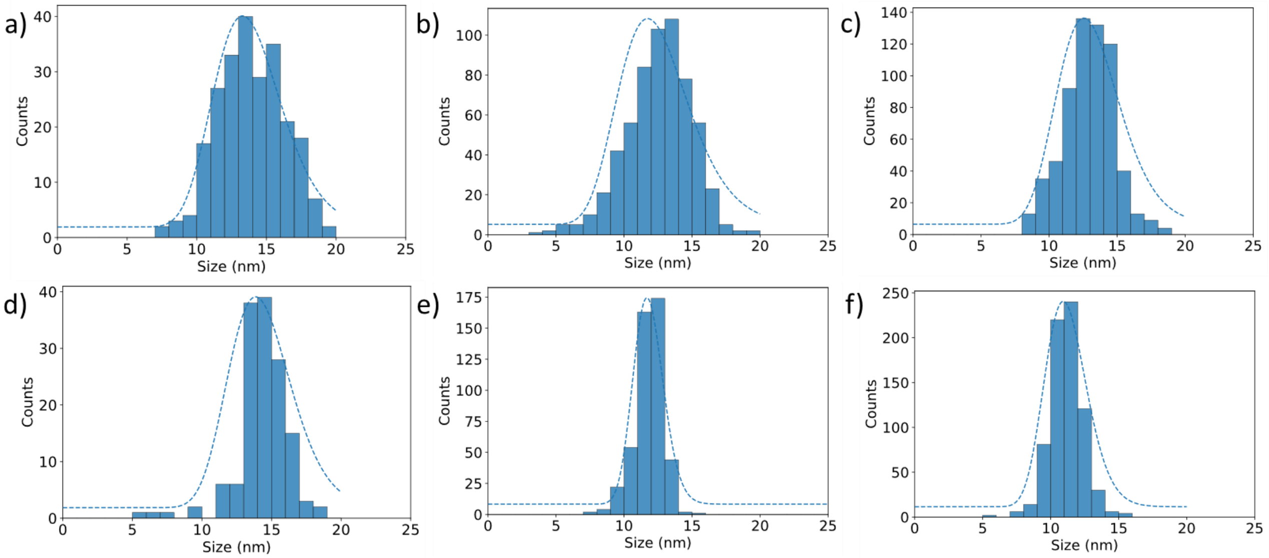

Figure S3: Size distributions of Pt-shell $Au_{1-x}Cu_x$-core samples reported on in this work for x = 0 (a), x = 0.19 (b), x = 0.28 (c), x = 0.38 (d), x = 0.55 (e) and x = 0. 77 (f). Nanoparticle sizes are based on at least 200 counted particles.

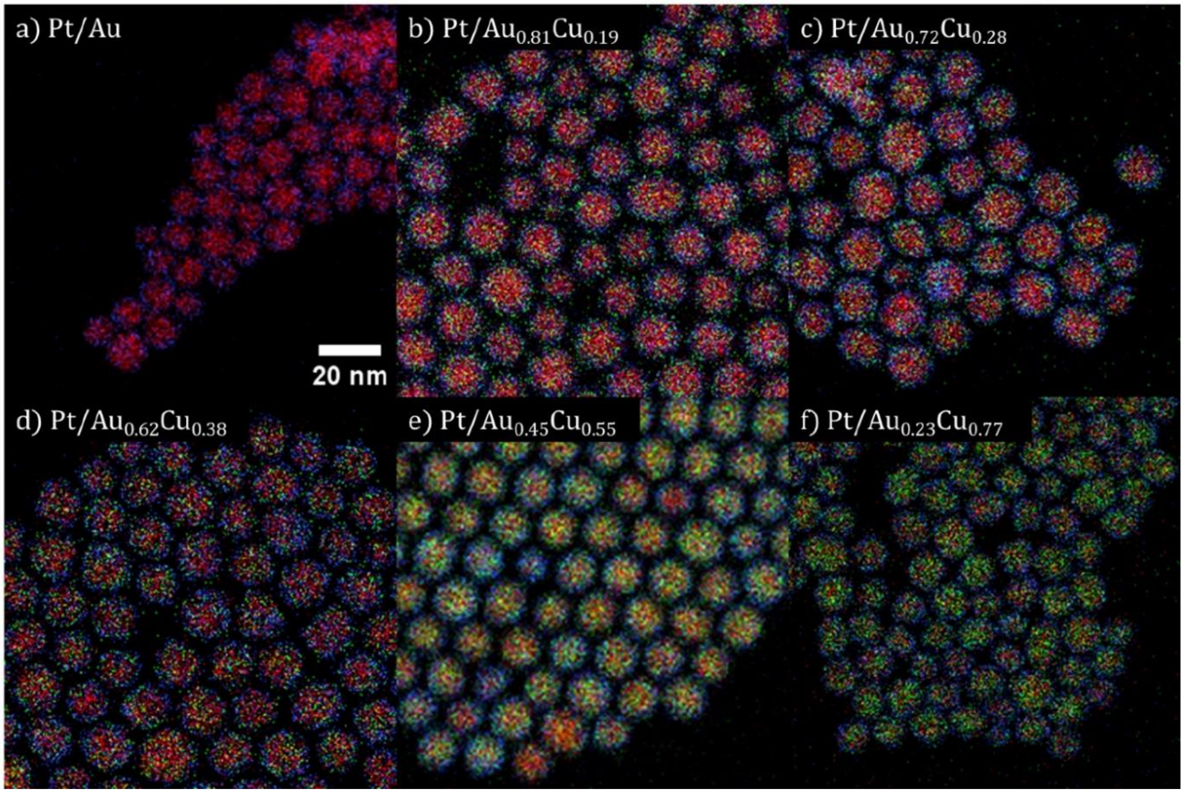

Figure S4: Energy-dispersive X-ray spectroscopy (EDX) maps for Pt-shell $Au_{1-x}Cu_x$-core with x = 0 (a), 0.19 (b), 0.28 (c), 0.38 (d), 0.55 (e) and 0.77 (f) as reported in the main text. Maps are slightly brightened to enhance visibility in the figure. Red, green & blue indicate gold-, copper- & platinum signals, respectively. The Pt signal was primarily located near the edge of the particles, whereas the Au and Cu signals were primarily observed in the core.

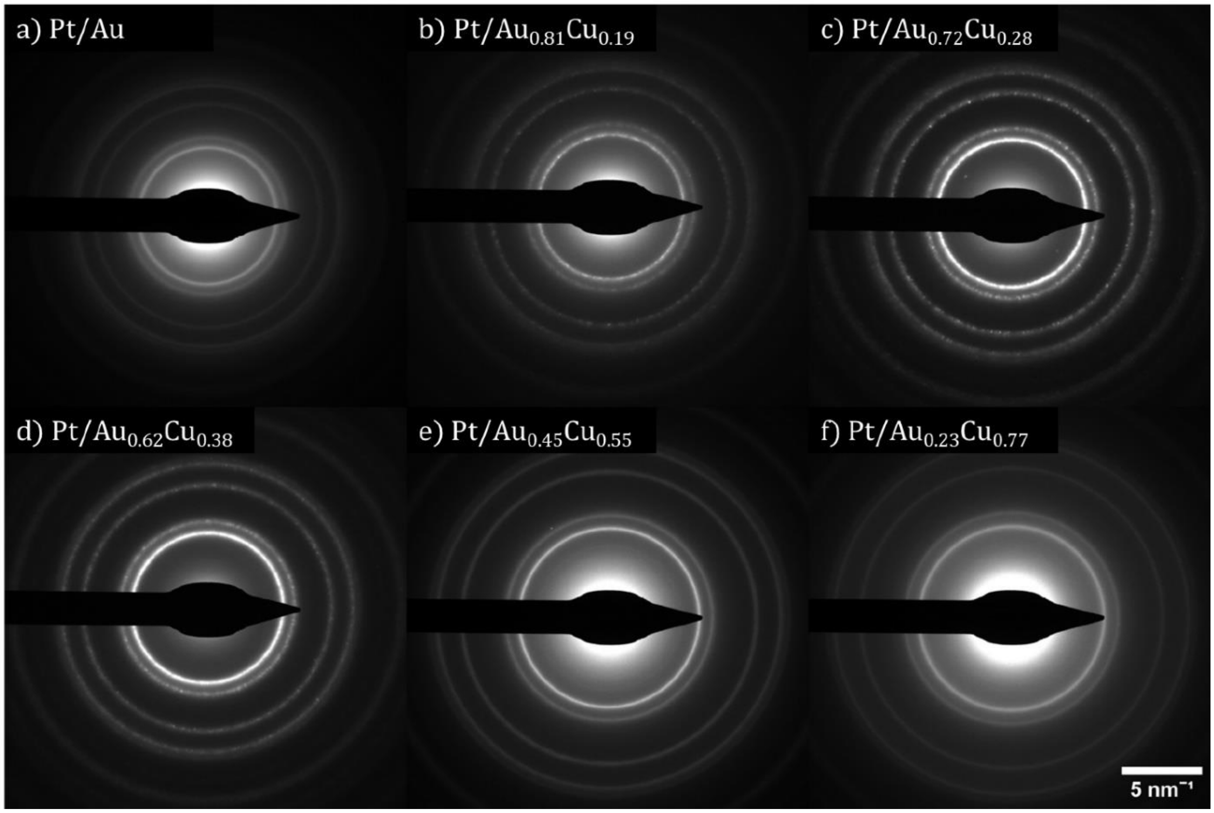

Figure S5: Electron diffraction patterns for Pt-shell $Au_{1-x}Cu_x$-core with x = 0 (a), 0.19 (b), 0.28 (c), 0.38 (d), 0.55 (e) and 0.77 (f) as as reported in the main text. Patterns are slightly brightened to enhance visibility in the figure. All images were analysed with CrysTBox

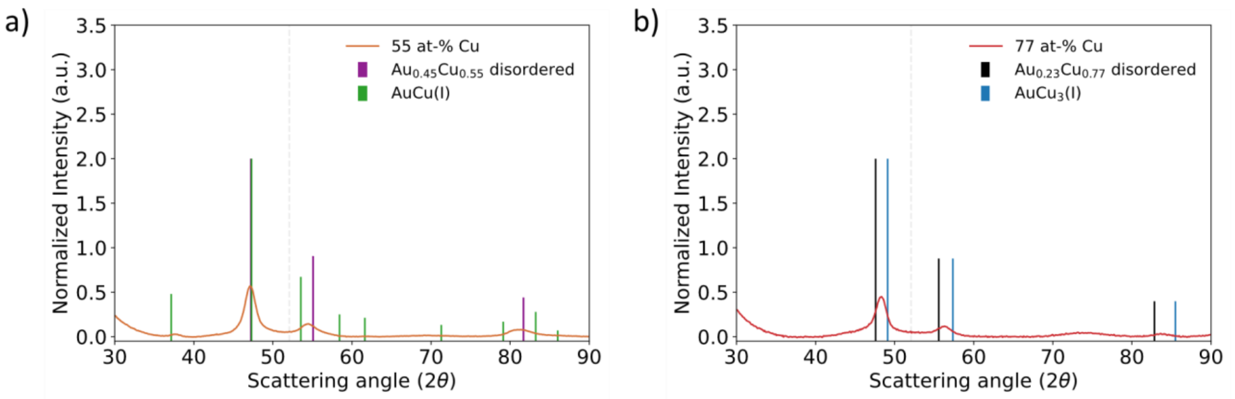

Figure S6: Assessment of intermetallic phase formation in Pt/$Au_{0.45}Cu_{0.55}$ (a) and Pt/$Au_{0.23}Cu_{0.77}$ (b). a) X-ray diffraction data for Pt/$Au_{0.45}Cu_{0.55}$ nanoparticles. Purple and green lines indicate diffraction pattern of reference disordered cubic AuCu (PDF:04-007-4433, corrected for composition) and tetragonal AuCu(I) phases (PDF: 01-089-2037), respectively. c) X-ray diffraction data for Pt/$Au_{0.23}Cu_{0.77}$ nanoparticles. Black and blue lines indicate diffraction pattern of reference disordered cubic AuCu3 (PDF: 01-072-5240, corrected for composition) and ordered cubic AuCu3(I) phases (PDF: 00-035-1357), respectively.

Observing atomically ordered $AuCu_3$(I). For a Pt/$Au_{0.23}Cu_{0.77}$ nanoparticle imaged with HRSTEM, an intermetallic $AuCu_3$(I) phase was observed. In Figure S7a, distinct dark and bright columns were observed. Brighter spots corresponded with Au-rich atomic columns, whereas darker spots corresponded to Cu-rich atomic columns to the Z-contrast scaling of STEM. These columns, were regularly spaced, indicating an intermetallic phase as shown in Figure S7b, where Au atoms occupied the corner positions of the unit cell whereas Cu atoms occupied the faces of the unit cell. In Figure S7c, an schematic of an extended crystal as imaged along the [1-10] zone axis is shown, which matches the real crystal shown in Figure S7a.

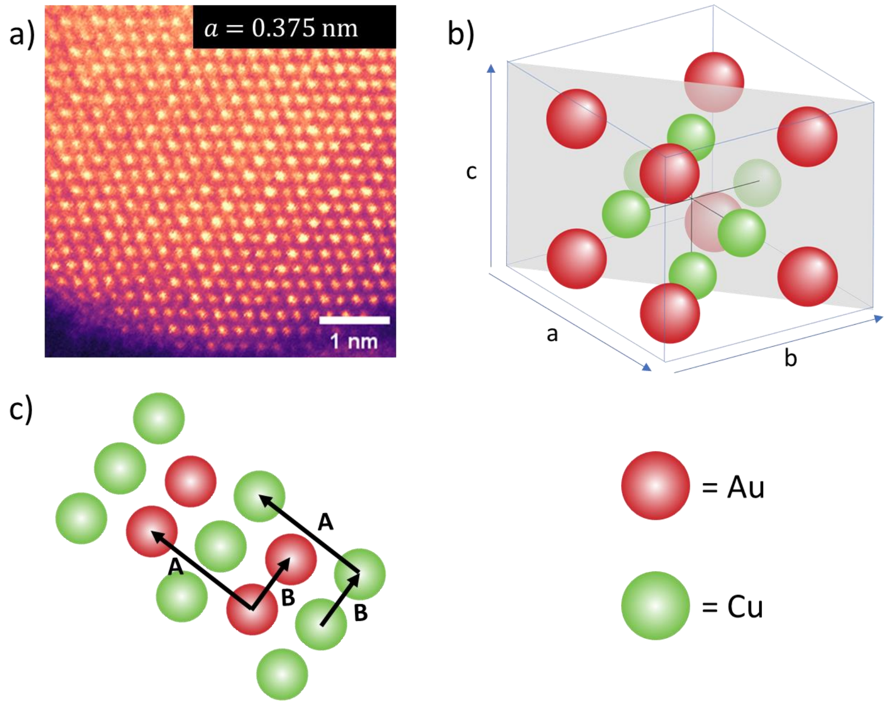

Figure S7: HRSTEM imaging of intermetallic $AuCu_3$(I) phase. a) HRSTEM image of a region of a Pt/$Au_{0.23}Cu_{0.77}$ nanoparticle imaged along the [1-10] zone axis. b) Unit cell of the $AuCu_3$(I) phase. Red spheres represent Au, and occupy the corners of the unit cell. Green spheres represent Cu, and occupy the faces of the unit cell. Grey rectangle represents the imaged crystal plane. c) Flattened unit cell from b) when imaged along the [1-10] zone axis as indicated by the grey rectangle. This corresponds well to the EM image shown above, which shows two distinct sets of atomic columns, one with darker spots, corresponding to Cu-rich atomic columns and one with brighter spots, corresponding to Au-rich atomic columns. Distances between Cu-rich columns and distances between Au-rich columns are equal (as represented by lines A & B), indicating an ordered cubic structure.

Determination of nanoparticle shape descriptors. In order to quantify particle shape and surface roughness, we used ImageJ software to determine three shape descriptors: circularity, aspect ratio and solidity. The circularity is a measure for how circular the nanoparticle is. Values of 1 represent a perfect circle, and values close to 0 represent elongated polygons. The aspect ratio corresponds to the symmetricity of the nanoparticle. Aspect ratios of 1 correspond to nanoparticles which are symmetric in size in 2 orthogonal dimensions, whereas larger aspect ratios correspond to particles which are elongated. The solidity describes the concavity of the nanoparticle, and is a measure for how solid the particle is. Values of zero represent very rough or non-solid surfaces, such as a nanostar. The corresponding equations are given below (S1-S3).

$$Circularity = \frac{4\pi * \text{area}}{perimeter\text{^}2} \tag{S1}$$

$$Aspect\ ratio = \frac{Length_{majoraxis}}{Length_{minoraxis}} \tag{S2}$$

$$Solidity = \frac{Area_{nanoparticle}}{Convex\ area_{nanoparticle}} \tag{S3}$$

The values for these shape factors are tabulated in Table S1 for the particle samples shown in Figure S2, and plotted in Figure S8. The particles do not show large differences in surface roughness, nor in aspect ratio. The circularity is slightly lower for the Pt/$Au_{0.81}Cu_{0.19}$ and Pt/$Au_{0.72}Cu_{0.28}$ sample.

Table S1: Overview of shape descriptors estimated with ImageJ software.

Sample	Size (nm)	Circularity	Aspect Ratio	Solidity
Pt/Au	13.9 ± 2.4	0.838 ± 0.093	1.181 ± 0.182	0.942 ± 0.076
Pt/$Au_{0.81}Cu_{0.19}$	12.6 ± 2.5	0.747 ± 0.073	1.106 ± 0.109	0.961 ± 0.075
Pt/$Au_{0.72}Cu_{0.28}$	13.2 ± 2.9	0.765 ± 0.110	1.116 ± 0.101	0.954 ± 0.122
Pt/$Au_{0.62}Cu_{0.38}$	14.4 ± 2.0	0.897 ± 0.079	1.087 ± 0.081	0.942 ± 0.076
Pt/$Au_{0.45}Cu_{0.55}$	11.8 ± 1.1	0.871 ± 0.033	1.100 ± 0.068	0.954 ± 0.026
Pt/$Au_{0.23}Cu_{0.77}$	11.3 ± 1.0	0.865 ± 0.053	1.153 ± 0.130	0.956 ± 0.050

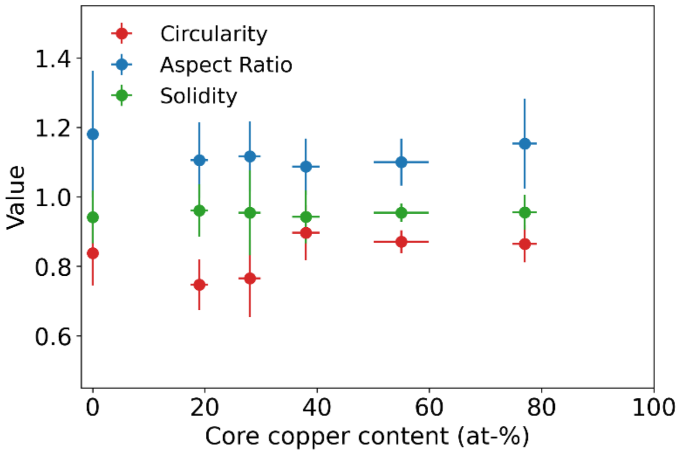

Figure S8: Shape descriptors for Pt-shell $Au_{1-x}Cu_x$-core with x = 0, 0.19, 0.28, 0.38, 0.55 and 0.77 reported in the main text. Shape descriptors were determined with ImageJ software.

REFERENCES

(1) van der Hoeven, J. E. S.; Deng, T.-S.; Albrecht, W.; Olthof, L. A.; van Huis, M. A.; de Jongh, P. E.; van Blaaderen, A. Structural Control over Bimetallic Core–Shell Nanorods for Surface-Enhanced Raman Spectroscopy. *ACS Omega* **2021**, *6* (10), 7034–7046. https://doi.org/10.1021/acsomega.0c06321.

(2) Klinger, M.; Jäger, A. Crystallographic Tool Box (CrysTBox): Automated Tools for Transmission Electron Microscopists and Crystallographers. *J Appl Crystallogr* **2015**, *48* (6), 2012–2018. https://doi.org/10.1107/S1600576715017252.
